# Molecular Cross-Talk between Integrins and Cadherins Leads to a Loss of Vascular Barrier Integrity during SARS-CoV-2 Infection

**DOI:** 10.3390/v14050891

**Published:** 2022-04-25

**Authors:** Danielle Nader, Steve W. Kerrigan

**Affiliations:** Cardiovascular Infection Research Group, School of Pharmacy and Biomolecular Sciences (PBS), RCSI University of Medicine and Health Sciences, 123 St. Stephen’s Green, D02 YN77 Dublin, Ireland; daniellenader@rcsi.com

**Keywords:** vascular dysfunction, SARS-CoV-2 variants of concern, Cilengitide, integrin αVβ3, RGD motif

## Abstract

The vascular barrier is heavily injured following SARS-CoV-2 infection and contributes enormously to life-threatening complications in COVID-19. This endothelial dysfunction is associated with the phlogistic phenomenon of cytokine storms, thrombotic complications, abnormal coagulation, hypoxemia, and multiple organ failure. The mechanisms surrounding COVID-19 associated endotheliitis have been widely attributed to ACE2-mediated pathways. However, integrins are emerging as possible receptor candidates for SARS-CoV-2, and their complex intracellular signaling events are essential for maintaining endothelial homeostasis. Here, we showed that the spike protein of SARS-CoV-2 depends on its RGD motif to drive barrier dysregulation by hijacking integrin αVβ3, expressed on human endothelial cells. This triggers the redistribution and internalization of major junction protein VE-Cadherin which leads to the barrier disruption phenotype. Both extracellular and intracellular inhibitors of integrin αVβ3 prevented these effects, similarly to the RGD-cyclic peptide compound Cilengitide, which suggests that the spike protein—through its RGD motif—binds to αVβ3 and elicits vascular leakage events. These findings support integrins as an additional receptor for SARS-CoV-2, particularly as integrin engagement can elucidate many of the adverse endothelial dysfunction events that stem from COVID-19.

## 1. Introduction

The interaction between the spike protein of SARS-coronavirus-2 (SARS-CoV-2) and endothelial cells has been widely demonstrated to be a critical driver in vascular dysregulation observed in COVID-19. Evidence in the literature describes a pattern of impaired vascular functionality following SARS-CoV-2 infection which involves the major endothelial adherens junction protein, VE-Cadherin [1]. The primary function of the vascular endothelium is to regulate systemic blood flow and maintain blood vessel wall permeability. It is made up of a highly adaptive single cell layer of cells held together by tight junction and adherens junction proteins. Tight junction proteins, such as occludins and claudins, control the permeability of solutes between blood and tissues, whereas adherens junction proteins, such as VE-Cadherin, control endothelial cell attachment and tight barrier formation [2]. Injury or insult to the vascular endothelium results in loss of barrier integrity leading to widespread microvascular hyperpermeability and secondary tissue damage. These published data demonstrate that significant disruption of junction proteins leads to reduced endothelial barrier integrity and subsequent monolayer permeability, elucidating the vast cardiovascular complications and septic shock experienced in severe COVID-19 [3]. Although the canonical ACE2 receptor has been implicated in driving this reaction, another well explored potential mechanism of action involves an integrin-mediated pathway [4]. Integrins are heterodimeric transmembrane proteins and key regulators of hemostasis, angiogenesis, proliferation, and inflammation. Activated through binding an RGD-containing ligand, integrins can control downstream signaling transduction cascades that tether VE-Cadherin at the cell junctions through RhoGTPase cycling [5,6]. SARS-CoV-2 spike protein contains an integrin-binding RGD motif that adheres to integrins αVβ3 and α5β1 on human pulmonary epithelial cells and human endothelial cells, where integrin antagonists Cilengitide and ATN-161 have demonstrated success in inhibiting this interaction in vitro and in vivo. These findings suggest integrin-targeted therapeutics may be of value in treating COVID-19 [1,7,8,9,10]. In this study, we investigated the intracellular signaling that occurs downstream of integrin engagement with the spike protein and whether targeting this receptor is sufficient in reducing the disease phenotype. 

## 2. Materials and Methods

### 2.1. Cell and Virus Culture Conditions

Primary-derived Human Aortic Endothelial Cells (HAoEC; Promocell C-12271) were maintained in Endothelial Cell Media MV (PromoCell) supplemented with 10,000 U/mL Penicillin and 100 mg/mL Streptomycin. Cells were subject to a physiological shear stress of 10 dynes/cm2. Human 2019-nCoV strain 2019-nCoV/Italy-INMI1 was obtained from the European Virus Archive Global (Ref. no: 008V-03893), and experiments were carried out using a Multiplicity of Infection (MOI) of 0.4.

### 2.2. ELISA

Interactions between recombinant spike proteins and integrins were performed as described previously [1]. Briefly, a 96-well microplate was coated with 25 ng of integrin αVβ3 (3050-AV, R&D Systems) overnight at 4 °C and blocked in 5% dry milk in 0.1% Tween 20-PBS. Anti-αVβ3 mAB (MAB1876-Z, 1:100), anti-β3 mAB (sc-46655, 1:100), Cilengitide (0.0005–0.05 µM), GLPG-0187 (0.05–10 µM), and spike proteins (40591-V08H41, 40591-V08H23, 50 nM) were added and washed. After incubation for 1 h with AlexaFluor 405-labeled spike protein antibodies (FAB105403V, 1:100), absorbance was measured at 405 nm. 

### 2.3. Endothelial Permeability and Immunofluorescence Assays

Endothelial barrier injury and VE-Cadherin expression were measured using transwell permeability assays and immunofluorescence, as described previously [1]. Briefly, HAoEC were grown to confluence on top chambers of inserts and infected with SARS-CoV-2 for 24 h. Cells were pre-treated for 30 min with Src and FAK inhibitors (SU6656, PF562271, 1 µM). Fluorescein isothiocyanate-dextran (250 ug/mL, 40 kDa, Sigma-Aldrich, Ireland) was added to the chambers, and fluorescent intensity was measured at 490/520 nm wavelengths. Cells grown on glass slides were infected and stained using anti-VE-Cadherin mouse monoclonal IgG1 antibody, conjugated to AlexaFluor 488 (F-8 sc-9989, 1:100), overlaid onto Fluoroshield mounting medium (ab104139). To measure internal VE-Cadherin, cells were acid-washed briefly following antibody staining. Cells were imaged using an AxioObserver Z1 microscope. 

### 2.4. Western Blot Analysis of VE-Cadherin

HAoEC were grown to confluency, pre-treated with Cilengitide (0.0005 µM) for 1 h, and infected with SARS-CoV-2 for 24 h. Scraped supernatants were collected in 150 µL of RIPA buffer. Proteins (10 µg) were loaded into an SDS-PAGE and stained using anti-VE-Cadherin mouse antibodies (F-8, sc-9989, 1:200) followed by anti-mouse secondary antibodies (sc-525405, 1:5000). GAPDH (ab-8245) was used as a loading control. 

## 3. Results

### 3.1. SARS-CoV-2 Variants of Concern Recognize Integrin αVβ3 through Conserved RGD Site

**Figure 1:**
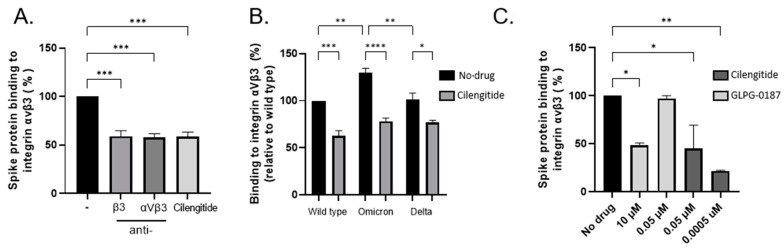
Wild type, Delta, and Omicron spike proteins bind integrin αVβ3 through its RGD site. (**A**) Binding of SARS-CoV-2 spike protein to recombinant integrin αVβ3 in an ELISA-based assay. Integrin-blocking antibodies αVβ3 and β3 inhibit the interaction between recombinant spike protein and αVβ3, similarly to Cilengitide (0.0005 µM) (One-way ANOVA, *** *p* < 0.001). (**B**) Effects of Cilengitide on variants of concern Delta and Omicron spike proteins binding to integrin αVβ3 (One-way ANOVA, * *p* < 0.05, ** *p* < 0.01, **** *p* < 0.0001). (**C**) Effects of broad-spectrum pan integrin inhibitor GLPG-0187 on spike protein binding to integrin αVβ3, compared to Cilengitide (One-way ANOVA). Values are mean ± S.E.M., *n* = 3.

In silico modelling of the spike protein RGD site (403–405) located with the receptor-binding domain (RBD) has predicted numerous points of contact with its putative integrin receptor, αVβ3 [1]. It has been proposed that select mutations within SARS-CoV-2 variants of concern enable a more wide-open RBD, thereby increasing the likelihood of host–virus recognition [11]. We performed ELISA-based assays to confirm the direct binding between integrin αVβ3 and spike protein of wild-type, Delta (B.1.617.2), and Omicron (B.1.1.529) in the presence or absence of the cyclic RGD peptide compound Cilengitide and neutralizing monoclonal antibodies which target the active site of the αVβ3 integrin or the β3 subunit. Cilengitide was successful in reducing the association between the integrin and spike protein, similarly to antibodies, revealing the interaction is likely RGD-dependent (Figure 1A,B). Comparatively, other integrin antagonists have demonstrated efficacy in reducing spike protein adherence to integrins such as GLPG-0187, a broad-spectrum pan integrin inhibitor [8,10]. Although successful at high concentrations, GLPG-0187 was unable to maintain sufficient blocking between the proteins, unlike Cilengitide which remained effective even at subnanomolar levels (Figure 1C). 

### 3.2. Spike Protein Causes VE-Cadherin Internalization Which Disrupts Vascular Permeability

**Figure 2:**
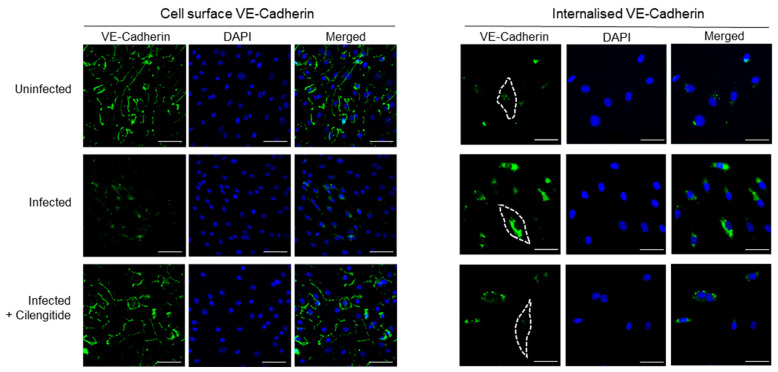
Vascular-Endothelial Cadherin is targeted by RGD site of SARS-CoV-2 spike protein to drive vascular dysfunction. Immunofluorescence images of a confluent human endothelial cell monolayer stained with VE-Cadherin and DAPI, measured for either cell-surface or internal VE-Cadherin. Dotted lines represent the endothelial cell border as visualized using DIC. Scale bars represent 10 µm.

Following spike protein engagement of integrins, reduced expression of some intercellular junction proteins (JAM-A and Connexin-43) has been detected in cerebral microvascular cells, alongside downregulation of the major adherens junction protein VE-Cadherin, which functions to mediate cell–cell adhesion [3]. Furthermore, evidence suggests SARS-CoV-2 spike protein directly induces this hyperpermeability through its RGD site, as treatment using the RGD peptide compound Cilengitide reduced inter-endothelial gaps and restored barrier function [1]. However, RGD-recognizing integrins are known to spatio-temporally coordinate the intracellular cycling of specific RhoGTPases to control VE-Cadherin without stimulating its downregulation [12]. To investigate this matter, we evaluated whether the endothelium could experience hyperpermeability through VE-Cadherin localization during SARS-CoV-2 infection. The in vitro barrier model composed of a confluent endothelial monolayer was exposed to SARS-CoV-2 cells for 24 h, with and without Cilengitide treatment. Cell-surface or external VE-Cadherin levels were significantly reduced following viral infection for 24 h, and blocking the RGD-binding site of integrins on host endothelial cells prevented this phenomenon (Figure 2). To measure non-surface bound VE-Cadherin, we utilized an acid-wash immunofluorescence staining protocol that enables the detection of internalized proteins. The amount of VE-Cadherin trafficked to intracellular compartments was significant in virally infected cells, revealing that intricate VE-Cadherin dynamics are likely involved in modulating vascular permeability during COVID-19. 

**Figure 3:**
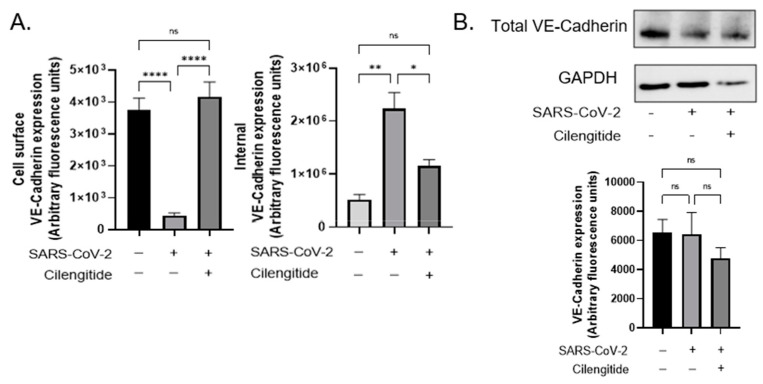
SARS-CoV-2 RGD motif triggers internalization of Vascular-Endothelial Cadherin. (**A**) Quantification of VE-Cadherin levels computed using ImageJ analysis, following background removal (* *p* < 0.05, ** *p* < 0.01, **** *p* < 0.0001, One-way ANOVA). (**B**) Western blot analysis performed on total VE-Cadherin expression in healthy and SARS-CoV-2 infected endothelial cells. Treated cells were incubated with 0.0005 µM Cilengitide for 1 h. Representative densitometry performed on Western blot (One-way ANOVA). Ns = not significant. Values are mean ± S.E.M., *n* = 3.

Treating the infected cells with the αVβ3 integrin antagonist Cilengitide reduced the amount of internal VE-Cadherin to nearly uninfected basal levels (Figure 3A, right panel). Our findings reveal that the spike protein binding to integrin αVβ3 directly triggers the integrin-mediated VE-Cadherin pathway in endothelial cells responsible for controlling vascular permeability. Moreover, the RGD site of the spike protein drives this pathway. Our data additionally revealed that VE-Cadherin levels were consistently stable overall in both healthy and infected vascular endothelial cells (Figure 1B).

### 3.3. Targeting Intracellular Integrin Pathways Can Prevent Vascular Dysregulation Following Infection

**Figure 4:**
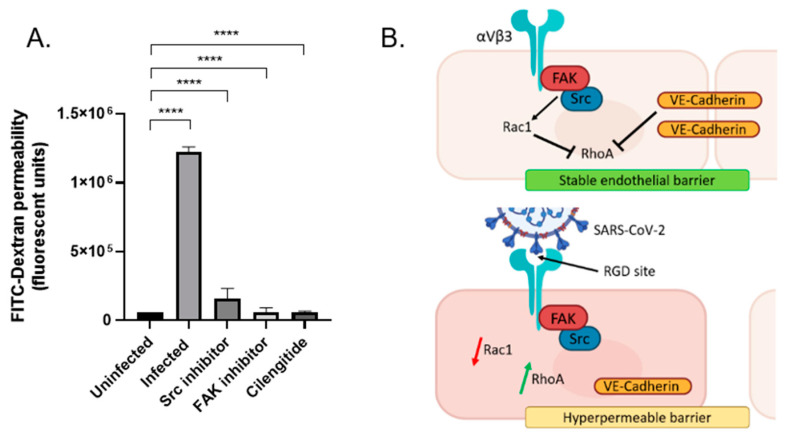
Vascular permeability induced by SARS-CoV-2 infection is mediated by endothelial integrin αVβ3. (**A**) Pharmacological inhibitors of Src (SU6656, 1 µM), FAK (PF562271, 1 µM), and integrin αVβ3 (Cilengitide, 0.0005 µM) incubated with SARS-CoV-2 infected endothelial cells. **** *p* < 0.0001, One-way ANOVA. Values are mean ± S.E.M., *n* = 3. (**B**) Schematic depiction of the proposed pathway of integrin engagement with spike protein and subsequent signaling cascade leading to the disease phenotype of hyperpermeability. Top panel portrays a healthy endothelial cell, where VE-Cadherin and Rac1 regulate and maintain low RhoA levels through FAK and Src signaling. Rac1 and RhoA signaling is tightly controlled via integrin engagement with an extracellular ligand. Bottom panel portrays an infected endothelial cell, where persistent integrin activation leads to overactive FAK and Src activity, resulting in faulty cycling between RhoA and Rac1. RhoA levels rise, leading to Cadherin phosphorylation via Src and FAK. Catenins, which confine VE-Cadherin at the endothelial junctions, cannot recognize phosphorylated VE-Cadherin which results in its internalization. This causes endothelial cells to pull apart and hyperpermeability to occur, promoting vascular leakage.

Focal adhesion kinase (FAK) and Proto-oncogene tyrosine-protein kinase Src (Src) are non-receptor tyrosine kinases that localize to the integrin β tail and are widely implicated in coordinating integrin signaling transduction in response to external stimuli such as adhering to an RGD ligand. Since these proteins regulate the RhoGTPases that control VE-Cadherin internalization, we investigated whether inhibiting these proteins associated with the integrin could also encourage similar events of vascular barrier protection as the integrin antagonist. Inhibition of FAK and Src prevented endothelial hyperpermeability in response to SARS-CoV-2 infection over 24 h. Furthermore, this reduction was comparable to Cilengitide (Figure 4A). 

## 4. Discussion

Clinical observations of viral endotheliitis, pulmonary thrombosis, hypoxia, edema, and acute cardiac injury in patients with severe COVID-19 are indicative of a dysfunctional endothelial barrier, which establishes it as a vascular disease [13]. The relationship between SARS-CoV-2 spike protein and its host receptor ACE2 has been well defined, and a dual-receptor mechanism has been proposed with another class of cell surface receptor, integrins. In particular, integrins αVβ3 and α5β1 recognize the RGD motif expressed by SARS-CoV-2, which mediates infection of epithelial and endothelial cells in vitro and in vivo [1,7,8,9,10]. Subsequently, inter-endothelial junction weakening and hyperpermeability have been observed, which likely elucidates the pulmonary and cardiovascular complications in COVID-19 [1,3,9]. Inhibiting spike protein attachment hinders this response [1,9]. Therefore, we sought to describe the nature underlying the loss of barrier integrity in SARS-CoV-2 infection. 

Our work has identified the downstream signaling transduction cascade that links integrins directly to the observations of vasculopathy in COVID-19. Firstly, the spike proteins of Delta and Omicron SARS-CoV-2 variants of concern are still highly recognized by integrin αVβ3 as they both retain the RGD site. Both Cilengitide and integrin neutralizing antibodies similarly blocked spike binding to integrins, revealing that this interaction is likely RGD-dependent. Other integrin antagonists such as GLPG-0187 have successfully displayed efficacy in reducing spike protein infection when used at high concentrations, confirming its involvement as a spike protein receptor [10]. However, when tested at similar concentrations to Cilengitide (0.05 µM), it failed to prevent attachment. This may be due to the broad-spectrum activity of GLPG-0187 compared to the highly specific affinity Cilengitide has towards αVβ3 (IC50 = 0.58 nm). Similar to the α5β1 antagonist ATN-161, Cilengitide has undergone clinical trials for the treatment of glioblastoma where it was greatly tolerated by patients due to its notable safety profile [14]. This has critical implications for COVID-19 treatment. Neutralizing antibodies recognize the ACE2 binding interface located on the spike protein surface (residues 437–507), and therefore mutations affecting receptor recognition often result in antibody evasion. Some in vivo and in vitro success has been observed using antibody cocktails to reduce viral load in SARS-CoV-2, particularly Etesevimab and Bamlanivimab combined therapy [15]. However, both antibodies were sensitive to mutations found in circulating variants of concern B.1.351 and B.1.617.2, and B.1.1.529 was partially or completely resistant to 100% of neutralizing monoclonal antibodies [16]. The RGD (403–405) motif is located within the spike receptor-binding domain and is conserved across >99% of variants [17]. As vaccine and immune-induced immunity is a key contributor to viral evolution, developing a compound that targets less immunodominant epitopes such as the RGD motif could be a more effective strategy against SARS-CoV-2 variants. 

We recently identified that intercellular proteins were involved in SARS-CoV-2 pathogenesis, and this likely corresponded to the hyperpermeability observed across the endothelium in COVID-19 [1]. The major adherens junction protein VE-Cadherin was directly impacted during viral infection, where it was notably missing from its expected occupancy at the cell–cell contacts. When endothelial cells typically undergo angiogenesis and cell migration is required, the Rho GTPases Rac1 and RhoA tightly regulate stress fiber formation, whose spatially coordinated activation is triggered by integrins [12]. Subsequently, VE-Cadherin can undergo translocation into intracellular compartments via clathrin-mediated endocytosis upon integrin activation and downstream transduction signaling involving the major focal adhesion proteins FAK and Src [5]. We propose that SARS-CoV-2 hijacks integrins via its RGD motif and controls its signaling cascade to command hyperpermeability. This ensures severe hypoxia and serum leakage, circulatory collapse and organ failure, which are key indicators of sepsis development. Critically, sepsis-related morbidity has been significantly attributed to COVID-19 deaths in both ICU and non-ICU patients. To explicate this matter, we evaluated the involvement of VE-Cadherin following spike protein infection. Although previous data suggest that VE-Cadherin was downregulated to some extent alongside other gap and tight junction proteins, here we showed that SARS-CoV-2 spike protein binding to integrin αVβ3 did not have any obvious effect on VE-Cadherin levels. However, infection dramatically altered VE-Cadherin organization by triggering its internalization, which led to the dysfunctional barrier phenotype. Furthermore, the spike protein of SARS-CoV-2 has been demonstrated to signal the upregulation of RhoA in infected venous endothelial cells by downregulating Rac1, which promoted hyperpermeability and leakage [9]. Following integrin ligation, VE-Cadherin is known to coordinate with Rac1 to inhibit RhoA from regulating cell spreading [18] This is consistent with our own findings, where we have found an association between αVβ3 and vascular permeability, a process tightly controlled by VE-Cadherin. 

Additionally, several tyrosine sites across the cytoplasmic tail of VE-Cadherin undergo phosphorylation via FAK and Src proteins, and elevated phosphorylation of Y658 and Y731 accounts for the majority of barrier breakdown [19]. Pharmacological inhibition of Src and its substrate FAK was effective in stabilizing VE-Cadherin at the cell surface due to the significantly reduced endothelial permeability during SARS-CoV-2 infection. However, it has been reported that halting Src-mediated phosphorylation of VE-Cadherin is not especially sufficient to fully repair the endothelial barrier, suggesting a myriad of intracellular protein inhibitors would be required to suspend this occurrence [20]. Therefore, we suggest that eliminating the initial contact between viral and host protein—such as by using integrin antagonists—would be more effective. 

These early findings speculate that integrin engagement with the RGD-containing spike protein triggers a signaling cascade through FAK and Src, resulting in the downregulation of Rac1, increase in RhoA, and subsequent internalization of VE-Cadherin. Although some VE-Cadherin downregulation has been documented to occur, plasma membrane-associated VE-Cadherin can translocate into intracellular compartments following integrin activation through the RGD site of the spike protein. As pools of endocytosed Cadherins get recycled back to the plasma membrane, this could elucidate the process of vascular recovery in patients that do not experience severe COVID-19, although sepsis events can still take place [4,12]. Although the canonical ACE2 receptor certainly plays a role in interacting with spike proteins and driving tissue inflammation and injury, integrins are predominantly responsible for controlling endothelial permeability. Therefore, we propose this is likely an integrin-exclusive pathway exploited by the RGD motif of the spike protein.

## 5. Conclusions

Altogether, we have shown that spike protein binding to integrin αVβ3 significantly impacts the integrity of the vascular barrier. Through its RGD site, SARS-CoV-2 effectively exploits downstream integrin signaling cascades to induce hyperpermeability, which elucidates the dysfunctional vascular phenotype in COVID-19. An αVβ3 integrin inhibitor, Cilengitide, has displayed promising results in blocking this event. These early findings suggest that removing the initial signal that triggers integrin activation is capable of reducing the downstream signaling cascades that regulate cellular hyperpermeability in COVID-19. Evidently, endothelial cells are critical players during viral infection, and delineation of the mechanisms surrounding vascular integrity is required for the development of therapies to counteract the pathogenesis of SARS-CoV-2.

## Data Availability

Supporting data can be supplied upon reasonable request.
